# Developing Fast Fluorescent Protein Voltage Sensors by Optimizing FRET Interactions

**DOI:** 10.1371/journal.pone.0141585

**Published:** 2015-11-20

**Authors:** Uhna Sung, Masoud Sepehri-Rad, Hong Hua Piao, Lei Jin, Thomas Hughes, Lawrence B. Cohen, Bradley J. Baker

**Affiliations:** 1 Center for Functional Connectomics, Korea Institute of Science & Technology, Seoul, Korea; 2 Department of Molecular and Cellular Physiology, Yale University School of Medicine, New Haven, CT, United States of America; 3 Department of Cell Biology and Neuroscience, Montana State University, Bozeman, MT, United States of America; University of California, Berkeley, UNITED STATES

## Abstract

FRET (Förster Resonance Energy Transfer)-based protein voltage sensors can be useful for monitoring neuronal activity *in vivo* because the ratio of signals between the donor and acceptor pair reduces common sources of noise such as heart beat artifacts. We improved the performance of FRET based genetically encoded Fluorescent Protein (FP) voltage sensors by optimizing the location of donor and acceptor FPs flanking the voltage sensitive domain of the *Ciona intestinalis* voltage sensitive phosphatase. First, we created 39 different “Nabi1” constructs by positioning the donor FP, UKG, at 8 different locations downstream of the voltage-sensing domain and the acceptor FP, mKO, at 6 positions upstream. Several of these combinations resulted in large voltage dependent signals and relatively fast kinetics. Nabi1 probes responded with signal size up to 11% ΔF/F for a 100 mV depolarization and fast response time constants both for signal activation (~2 ms) and signal decay (~3 ms). We improved expression in neuronal cells by replacing the mKO and UKG FRET pair with Clover (donor FP) and mRuby2 (acceptor FP) to create Nabi2 probes. Nabi2 probes also had large signals and relatively fast time constants in HEK293 cells. In primary neuronal culture, a Nabi2 probe was able to differentiate individual action potentials at 45 Hz.

## Introduction

Developing genetically encoded fluorescent voltage sensitive probes can provide tools for optical detection of neural information at the level of individual neuron types in an interconnected neural network. Voltage sensitive organic dyes detect changes in membrane potential with a signal size linearly dependent on voltage and with a signal speed that is fast compared to the rise time of an action potential [1]. However, the use of organic dyes is limited due to nonspecificity and low accessibility of cell types deeper than 250 μm from the surface. In contrast, fluorescent protein (FP) voltage sensors can be targeted to individual cell types deep within the mammalian brain.

Microbial rhodopsin-based FP voltage sensors such as Arch generate signals with fast kinetics and a large fractional fluorescence change [2–4]. However, their use for *in vivo* imaging can be limited by their very dim fluorescence that can be obscured by the intrinsic fluorescence. In addition, they require intense illumination for imaging. In contrast, FP voltage sensors with FPs such as super-ecliptic pHluorin [5] are 100 times brighter than rhodopsin-based voltage probes and therefore more likely to function well in *in vivo* measurements from mammalian brains [6]. Recently developed voltage probes with the voltage sensitive domain from voltage sensitive phosphatases show increased dynamic range and fast kinetics in responding to action potentials [5, 7–10]. FRET-opsin (electrochromic FRET) voltage probes combine rhodopsin-based voltage probes with conventional FPs to overcome dim fluorescence [11, 12]. However, these FRET probes are not ratiometric.

Developing FP voltage sensors that utilize FRET between two FPs can be useful for *in vivo* imaging because the ratio of the FRET signals reduces common source noise such as heartbeat and breathing artifacts. The performance of FRET based voltage sensors has lagged behind those of single-FP voltage sensors in terms of signal size and kinetics [5, 9, 10]. None of the recently described FRET based probes produces signals capable of resolving fast trains of action potentials in neurons [8, 13, 14].

The voltage sensing domain of the *Ciona* voltage sensitive phosphatase is known to undergo voltage dependent conformational changes [15–17]. The recently described crystal structure of the up and down states of the *Ciona* voltage sensing domain reveals the movement of the S4 helix accompanied by a rearrangement of S1 and S3 helices to up or down conformations [17]. These movements could change the distance and/or orientation between donor and acceptor chromophores attached to the voltage sensitive domain, leading to changes in FRET efficiency. The location of the FPs in the voltage sensitive domain can affect the membrane targeting and voltage responses [8, 13, 18]. In VSFP and Mermaid [7, 18], donor and acceptor FPs are inserted in tandem downstream of the S4. A variant of Mermaid with insertion of 2 FPs in tandem before the S1 demonstrated that conformational changes in the N-terminus can also influence FRET responses [19]. Positioning donor and acceptor FPs on the opposite sides of the S1-S4 domain as in VSFP Butterfly1.2 [13] and Mermaid2 [8] improved the signal amplitude or kinetics compared to versions with FPs in tandem. FP voltage sensors with FPs in a VSFP Butterfly1.2 configuration may result in greater movements of the FPs compared to sensors with FPs in tandem.

Whereas recent successes of creating novel voltage probes were achieved by rational design and mutagenesis [9, 10], we used an empirical approach to uncover optimal locations of FPs located in the flanking regions of the *Ciona* voltage-sensing domain. We aimed at improvements in signal size, kinetics, and a diverse range of voltages over which the fluorescent signals occur (V_1/2_). A combinatorial library of constructs, named “Nabi” (“butterfly” in Korean), were generated with one FP at 6 different locations in the N-terminus and the other FP at 8 different locations downstream of S4 in the C-terminus. We created two groups of FRET based voltage sensors, Nabi1 with UKG and mKO as the FRET pair [18], and Nabi2 with Clover and mRuby2 as the pair [20]. Each Nabi1 and Nabi2 probe displayed voltage dependent fluorescence changes with unique signal kinetics and size. Many Nabi probes showed signals with improved signal size and faster kinetics than previously described butterfly FRET based voltage sensors such as VSFP Butterfly 1.2 [13] and VSFP-CR [20].

## Materials and Methods

### Molecular biology

We created the Nabi1 constructs by PCR amplification and ligation independent cloning (InFusion, Clontech). First, PCR was used to combine fragments of the voltage sensing domain and either the donor or acceptor fluorescent protein to create full-length fusion proteins carrying only one FP. These were cloned into pUB2.1, which is a CMV expression plasmid fitted with a ccdB negative selection cassette that eliminates vector-only background (Addgene plasmid 40728: pUB2.1). Primers complementary to the start of the coding region and a reverse primer complementary to the region encoding the S4 domain were then used in a second set of PCR reactions to generate products encoding the first half of the fusion protein, and a forward primer complementary to the S4 region was paired to a reverse primer at the stop codon to generate PCR products that encoded the second half of the protein including the FP. These PCR products were then combinatorially cloned into pUB2.1 to generate the 39 Nabi1 constructs (Table 1). Because the UKG and mKO FRET pair did not express well in neurons [21], eight combinations with promising optical signals had their fluorescent proteins replaced with the Clover/mRuby2 FRET pair [20]. These constructs, designated Nabi2 were made by replacing corresponding mKO and UKG of Nabi1 probes with Clover (Addgene plasmid 40259) and mRuby2 (Addgene plasmid 40260). Attempts to remove the unstructured region beyond the FP barrel of mRuby2 led to poor folding (Lin MZ, personal communication). The unstructured region at the C-terminus of mRuby2 can act as a linker that increases the physical distance between the FRET pairs. On the other hand, the carboxyl terminus of the unstructured region of Clover can be truncated [20]. Therefore, we placed Clover amino acids 1–228 at the position of mKO and mRuby2 amino acids 1–237 at the position of UKG. This led to a switching of the positions of donor and acceptor FPs between Nabi1 and Nabi2. In order to fuse FPs without modification in the CiVSP backbone between Nabi1 and Nabi2, two-step PCR reactions were employed for the replacement. Insertion sites of FPs and sequences of Nabi1 and Nabi2 were verified by sequencing. We also made measurements in HEK293 cells with VSFP-CR [20] (Addgene 40257) and VSFP-Butterfly1.2 [13].

**Table 1:** Structure and voltage responses of Nabi1 probes. Nabi1 is composed of the N-terminus of the *Ciona* voltage sensitive domain, mKO (1–218 amino acids), the S1-S4 of the voltage sensitive domain, and UKG (1–224 amino acids), followed by a stop codon. Nabi1 probes were evaluated by using 100 ms voltage steps from a -70 mV holding potential to -170 mV, -20 mV, +30 mV, and +130 mV or to -120 mV, -20 mV, +30 mV, +80mV, and +130 mV in transiently transfected HEK293 cells. We tested 3–21 cells for each Nabi1 probe. Averaged optical traces from 16 trials were analyzed. ΔF/F was calculated for 100 mv depolarizations by taking the average of optical signals from the tested cells for each probe. ΔF/F_max_ was the largest value of the optical responses observed during any depolarizing voltage step. V_1/2_ (membrane potential at half maximum ΔF/F) was calculated from optical signals of all tested cells. The time constants were calculated using a double exponential fitting of 1–6 representative donor traces for each probe.

	Nabi1 protein structure				ΔF/F at 100mV (acceptor, donor)	ΔF/F_max_ (acceptor, donor)	V_1/2_	τ1 on	τ2 on	τ1 off	τ2 off
Nabi1	CiVSP amino acids	mKO amino acids	CiVSP amino acids	UKG amino acids							
					%	%	mV	ms	ms	ms	ms
Nabi1.103	1–42	1–218	43–243	1–224	1, -2	4, -2	-2	5	27	23	79
Nabi1.183	1–42	1–218	43–245	1–224	3, -3	7, -8	-31	13	52	23	110
Nabi1.82^1^	1–42	1–218	96–245	1–224	4, -4	5, -7	-37	4	51	4	83
Nabi1.201	1–42	1–218	43–246	1–224	4, -3	7, -7	-28	12	37	23	104
Nabi1.86	1–42	1–218	43–249	1–224	4, -4	7, -8	-35	11	48	18	92
Nabi1.207	1–42	1–218	43–252	1–224	2, -3	3, -4	-60	8	45	21	219
Nabi1.96	1–42	1–218	43–257	1–224	1, -3	6, -5	-61	5	39	20	330
Nabi1.98	1–42	1–218	43–259	1–224	2, -3	9, -8	-63	6	37	15	232
Nabi1.102^2^	1–42	1–218	43–259	1–224	3, -3	6, -5	-84	7	18	11	185
Nabi1.170	1–53	1–218	54–245	1–224	3, -3	5, -5	-27	6	40	18	134
Nabi1.171	1–53	1–218	54–246	1–224	4, -3	8, -8	-30	14	45	20	67
Nabi1.174	1–53	1–218	54–249	1–224	3, -2	7, -5	-50	7	42	24	135
Nabi1.169	1–53	1–218	54–252	1–224	3, -3	4, -5	-40	3	11	9	96
Nabi1.90	1–53	1–218	54–259	1–224	2, -4	5, -4	-60	4	27	13	138
Nabi1.213	1–84	1–218	85–245	1–224	6, -4	12, -13	-13	7	98	12	59
Nabi1.216	1–84	1–218	85–246	1–224	5, -3	7, -11	-7	2	68	5	62
Nabi1.220	1–84	1–218	85–249	1–224	6, -4	9, -17	-33	4	42	11	85
Nabi1.223	1–84	1–218	85–252	1–224	4, -4	11, -11	-25	12	73	8	63
Nabi1.66	1–84	1–218	85–257	1–224	3, -4	8, -8	-58	2	27	9	77
Nabi1.58	1–84	1–218	85–259	1–224	3, -3	6, -6	-48	4	21	13	66
Nabi1.30	1–90	1–218	91–241	1–224	3, -3	6, -8	-22	3	107	3	49
Nabi1.226	1–90	1–218	91–245	1–224	5, -3	9, -10	12	4	193	4	47
Nabi1.125	1–90	1–218	91–246	1–224	3, -4	8, -7	-6	3	73	4	51
Nabi1.187	1–90	1–218	91–249	1–224	4, -3	9, -7	-30	2	30	11	41
Nabi1.104	1–90	1–218	91–252	1–224	3, -4	10, -8	-21	3	38	3	48
Nabi1.106	1–90	1–218	91–257	1–224	5, -5	9, -10	-61	2	20	11	71
Nabi1.131	1–90	1–218	91–259	1–224	4, -4	9, -8	-48	2	27	8	47
Nabi1.244	1–95	1–218	96–245	1–224	2, -2	8, -8	28	4	56	4	97
Nabi1.242	1–95	1–218	96–246	1–224	4, -3	13, -16	16	5	56	7	72
Nabi1.243^3^	1–95	1–218	96–246	1–224	2, -2	12, -10	53	2	31	11	45
Nabi1.252	1–95	1–218	96–249	1–224	4, -3	7, -10	16	3	46	9	49
Nabi1.137	1–95	1–218	96–257	1–224	3, -3	5, -5	-62	2	52	7	86
Nabi1.142	1–95	1–218	96–259	1–224	3, -3	4, -5	-62	2	30	9	77
Nabi1.41	1–100	1–218	101–243	1–224	1, -2	3, -2	-23	4	55	17	86
Nabi1.149	1–100	1–218	101–246	1–224	1, -1	3, -2	9	4	91	4	46
Nabi1.49	1–100	1–218	101–249	1–224	3, -3	4, -5	-20	2	36	5	44
Nabi1.154	1–100	1–218	101–257	1–224	3, -3	6, -6	-48	4	29	9	55
Nabi1.152^4^	1–100	1–218	101–257	1–224	4, -4	6, -5	-62	2	29	6	52
Nabi1.141	1–100	1–218	101–259	1–224	3, -4	8, -9	-53	4	54	10	113

^1^Nabi1.82 has a deletion from 43^th^ to 95^th^ amino acids of the *Ciona* voltage sensitive domain.

^2^Nabi1.102 has a P to L substitution at 43^th^ amino acid of the *Ciona* voltage sensitive domain.

^3^In Nabi1.243, UKG is followed by amino acids 247–576 of the *Ciona* phosphatase domain.

^4^Nabi1.152 has a G to D substitution at 101^th^ amino acid of the *Ciona* voltage sensitive domain.

### Cell culture, transfection

HEK 293 cells were maintained in Dulbecco’s Modified Eagle Medium with high glucose supplemented with 10% fetal bovine serum in an incubator at 37°C under air with 5% CO_2_. Cells were plated on coverslips coated with poly-L-lysine and transiently transfected with DNA in a 2 cm^2^ dish (one well of a 24 well plate). Transfection was performed by using Lipofectamine 2000 (Invitrogen). Hippocampal neurons were isolated from E18 mouse embryos. Pregnant mice (C57BL/6; Koatech) were sacrificed by rapid decapitation. Mice were handled by the procedures reviewed and approved by the Institutional Animal Care and Use Committee of Yale University and the Committee on the Ethics of Animal Experiments of KIST (Korea Institute of Science and Technology), and were carried out in accordance with the *Guide for the Care and Use of Laboratory Animals* as adopted and promulgated by the National Institutes of Health. Every effort was made to minimize animal discomfort or suffering, and the number of animals used. Neuronal culture was maintained in Neuro basal medium with 0.5 mM Glutamax-I and 1 ml of B-27 supplement (Invitrogen) per 50 ml of cultured medium. Seven to ten days after cell isolation, transient transfection was accomplished by using 2 μg DNA per 35 mm dish or 0.4 μg per 12 mm coverslip in a 24-well plate by using Lipofectamine 2000 (5 μl per 35 mm dish or 1 μl per 12 mm coverslip) or by using the Ca^2+^ phosphate precipitation method with Calphos mammalian transfection kit (Clonetech, TaKaRa) [9]. The neurons were used for experiments 1–3 days after transfection.

### Electrophysiology

Electrophysiological recordings from HEK293 cells were performed in a perfusion chamber with the bath temperature kept at 33°C by a temperature controller. The bathing solution was KRH (Krebs-Ringers HEPES) solution (120 mM NaCl, 4.7 mM KCl, 1.2 mM KH_2_PO_4_, 1.2 mM MgSO_4_, 10 mM HEPES, 2.2 mM CaCl_2_, and 1.8 mg/ ml D-glucose, pH 7.4). We used a 3–5 MΩ glass patch pipettes (capillary tubing with 1.5/0.75 mm OD/ID from World Precision Instruments, FL) that were pulled on a P-97 Flaming/ Brown type micropipette puller (Sutter Instrument Company, CA). The pipette solution for HEK293 cells contained 120 mM K-aspartate, 4 mM NaCl, 4 mM MgCl_2_, 1mM CaCl_2_, 10 mM EGTA, 3 mM Na_2_ATP and 5 mM HEPES, pH 7.2. The pipette solution for neuron recordings contained 120 mM K-gluconate, 3 mM KCl, 7 mM NaCl, 4 mM Mg-ATP, 0.3 mM Na-GTP, 20 mM HEPES and 14 mM Tris-phosphocreatin, pH adjusted with KOH to pH 7.3 [22] or was same as the pipette solution for HEK293 cells. Voltage-clamp recordings in the whole-cell configuration were performed using a Patch Clamp PC-505B amplifier (Warner Instruments, CT) or an EPC10 USB Patch Clamp amplifier (HEKA Electronik, Germany). We stepped membrane potential from a holding potential at -70 mV to -170 mV, -20 mV, +30 mV, and +130 mV; or from -70 mV to -120 mV, -20 mV, +30 mV, +80mV, and +130 mV. Cultured hippocampal neurons were recorded in current clamp mode using a 2 ms current injection to evoke a single action potential or 200 ms injections to evoke a train of action potentials. We calculated [23] the liquid junction potential for the pipette solution of 120 mM K-gluconate, 3 mM KCl, 7 mM NaCl, 4 mM Mg-ATP, 0.3 mM Na-GTP, 20 mM HEPES and 14 mM Tris-phosphocreatin pH7.3 to be 16 mV. We calculated the liquid junction potential for the pipette solution of 120 mM K-aspartate, 4 mM NaCl, 4 mM MgCl_2_, 1mM CaCl_2_, 10 mM EGTA, 3 mM Na_2_ATP and 5 mM HEPES, pH 7.2 to be 13 mV and measured it to be 16 mV. The microelectrode data was not corrected for liquid junction potentials.

### Wide field imaging

Whole-cell patch clamped HEK293 and primary neuronal cells on a 0.08–0.13 mm thick cover slip were imaged on an Olympus IX71 inverted microscope (Olympus, Japan) using an Olympus UPLANSAPO 60x/1.35 NA oil immersion objective and a XBP 75 W/2 OFR Xenon short arc lamp (OSRAM, MI) with a stabilized power supply (Cairn Research, UK). Illumination intensity was 2.5 mW mm^-2^. For imaging of Nabi1 probes with the mKO/UKG pair we used a 445/20 nm excitation filter, a 458 nm dichroic mirror in the microscope, followed by a Splitter (Optosplit II; Cairn Research) with a 560 nm dichroic, and 510/84 nm and 580/60 nm emission filters (Semrock, NY). For imaging of Nabi2 and VSFP-CR with the Clover/mRuby2 pair we used a 475/23 nm excitation filter and a 495 nm dichroic mirror on the microscope, followed by a 560 nm dichroic, and 520/40 and 645/75 emission filters (Semrock) in the splitter. For imaging of VSFP-Butterfly1.2 with mCitrine/mKate pair we used a 500/24 nm excitation filter, a 520 nm dichroic mirror, and 550/60 and 645/75 nm emission filters. The fluorescence images were demagnified by an Optem^®^ zoom system A45699 (Qioptiq LINOS Inc. NY) and projected onto a NeuroCCD-SMQ camera (RedShirtImaging, GA) or onto a FastCMOS-128x camera (RedShirtImaging) controlled by NeuroPlex software (RedShirtImaging). The images were recorded at a frame rate of 1000 fps with the NeuroCCD-SMQ camera or 500 fps with the FastCMOS-128x camera. For imaging of neuronal cultures particularly for the data of stimulated action potential, we used a Nikon Eclipse E6000FN upright microscope with a water immersion objective, Nikon Fluor 60×/1.00 N.A. (Nikon, NY). A 50 mW MLL-FN-473 nm laser (Changchun New Industries Optoelectronics Tech. Co., Ltd., China) light was transmitted into the microscope by a multi-mode fiber coupler (Siskiyou, OR), a quartz light guide and an Achromatic EPI-Fluorescence Condenser (Till Photonics, NY). The filters mounted on an OptoSplit II LS Image Splitter (Cairn) were dichroic mirror RR560-Di01-25x36 (Semrock), and emission filters FF01-534/42 (Semrock) and RG610 (Schott). All neuronal images were recorded at a frame rate of 1000 fps with the NeuroCCD-SMQ camera.

### Confocal imaging

Confocal images of Nabi2 transfected neurons were obtained with a Zeiss 780 LSM (Carl Zeiss AG, Germany) confocal laser scanning microscope using a Plan-Apochromat 63×/1.40 Oil DIC M27 objective. Chromophore excitation was by a 488 nm wavelength Argon laser or a 561 nm solid state laser. Dichroic beam splitters were MBS 488/561/633 and MBS 458/561, and emission filters were 493-553nm and 566–685 nm. Image acquisition and processing were carried out with Zeiss Zen 2012 software.

### Image analysis, data processing, and data presentation

NeuroPlex software was used to view the image sequences and output optical and electrophysiological recordings. The traces were the spatial average of the output of all of the pixels receiving light from the patched cell. Signal traces of Nabi1, Nabi2, and VSFP-CR shown in figures were from single trials. The signal trace of VSFP-Butterfly1.2 was shown with the average of 16 trials. Averaged traces of 16 trials were used for all analyses including ΔF/F, time constants, and V_1/2_. The values of ΔF/F are the averages of signals from individual HEK293 cells for a 100 mV depolarization. Table 1 also lists the largest signal size of donor and acceptor observed from any cell during the series of voltage steps. The time constants for a 100 mV depolarization are the averages calculated from 1–6 representative traces.

For the calculation of % ΔF/F, the dark image was subtracted from all frames, the average of a region of interest in each frame during a voltage step (F) was subtracted from the average of the region taken from ten frames prior to the event of interest (F_0_), and then this value was divided by F_0_; the calculation was [% ΔF/F = ((F–F_0_) / F_0_)*100]. Traces were imported into Origin 8.6 (OriginLab, MA) for the analysis of time constants, (the contribution of τ1 and τ2), FWHM (full-width at half maximum) and V_1/2_ of the signals. The probe dynamics were fit with either a single exponential equation [y = A1*exp(-x/t1) + y0] or a double exponential equation [y = y0 + A1*exp(-(x-x0)/t1) + A2*exp(-(x-x0)/t2)] where A1 and A2 are amplitudes, and τ1 and τ2 are time constants in ms. The signal *vs* voltage relationships were fit with the Boltzmann equation [y = A2+(A1−A2)/(1+ exp(x−x_1/2_)/s)], where x_1/2_ is V_1/2_ (the membrane potential in mV at half maximal ΔF/F), and s is the slope (Origin 8.6). V_1/2_ in Table 1 was estimated by assuming a linear relationship of optical signals between voltage steps on either side of the half maximal ΔF/F. Statistical analysis was performed using Origin 8.6, Prism 6 (Graphpad Software Inc., CA), or Excel (Microsoft, WA). The data are presented as mean ± SEM (the standard error of the mean).

### Estimation of the linker sequences of the FPs in Nabi1 and Nabi2

Sequence alignment of FPs was conducted by using NCBI BLAST, EMBL-EBI Clustal Omega, and by manual inspection. The amino acid sequences of linkers and β-strands of mKO, UKG, Clover, and mRuby2 were deduced based on protein sequence alignment and a comparison with the crystal structure of GFP [24].

## Results

### Nabi1 FP voltage sensors

**Fig 1:**
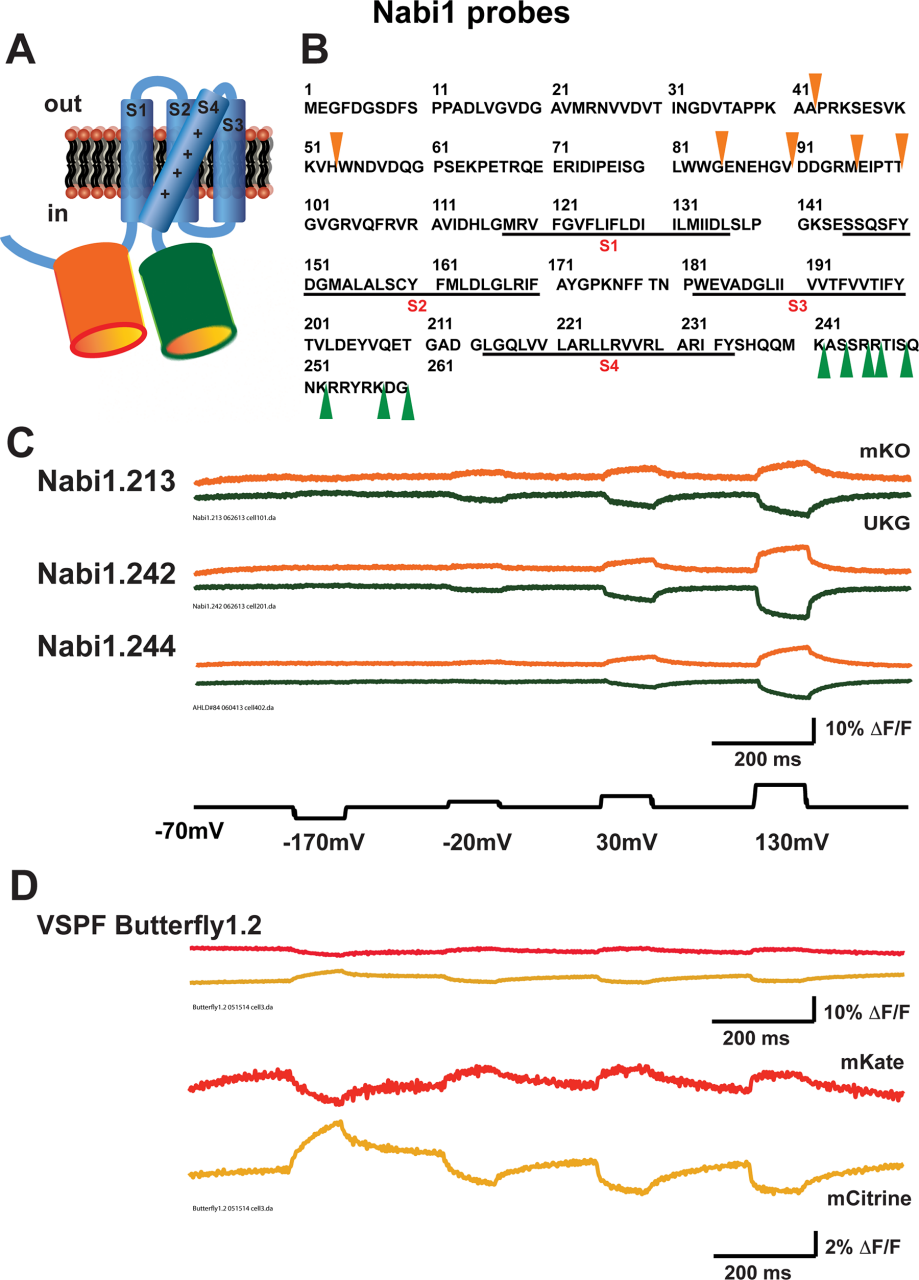
The structure and optical responses of Nabi1 probes to changes of membrane potential in transiently transfected HEK293 cells. **A.** Nabi1 probes contain mKO (orange) and UKG (green) as the FRET pair at flanking regions of the *Ciona* voltage sensitive domain. **B.** The sequence of the *Ciona* voltage sensitive domain (1^st^ to 259^th^ amino acids). The transmembrane domains S1-S4 are underlined. mKO was inserted at one of 6 locations indicated by orange arrowheads. UKG was inserted at one of 8 locations indicated by green arrowheads. **C.** Representative donor (UKG, green) and acceptor (mKO, orange) signals of 3 selected Nabi1 probes. Traces from single trials of Nabi1.213 (FPs at 84^th^ and 245^th^ amino acids), Nabi1.242 (FPs at 95^th^ and 246^th^), and Nabi1.244 (FPs at 95^th^ and 245^th^) are shown without temporal filtering. **D.** Representative donor (mCitrine, yellow) and acceptor (mKate, red) signals of VSFP Butterfly1.2 [13]. The traces are the average of 16 trials without temporal filtering and shown using scale bars of ΔF/F of 10% and 2%. The same voltage steps as in **C** were used for VSFP Butterfly1.2.

Our strategy to improve FP voltage sensors was to compare multiple locations for the donor and acceptor FPs in the backbone voltage sensitive domain. The Nabi1 constructs were designed with the insertion of UKG and mKO as the FRET pair at different locations in the cytoplasmic regions surrounding the *Ciona* voltage sensitive domain (Fig 1A and 1B). For insertion of UKG (green arrowheads in Fig 1B), we chose 8 sites downstream at the carboxyl end of S4. For insertion of mKO at the N-terminus (orange arrowheads in Fig 1B), we referred to previous reports [13, 19]. When a FP was inserted too close to the S1, membrane targeting of the *Ciona* voltage sensitive domain -based sensors were impaired [13]. When a FP was inserted before the 80^th^ amino acid of the voltage sensitive domain, voltage responses of the probes were reduced [19]. We selected four evenly spaced sites (84^th^, 90^th^, 95^th^, and 100^th^) between 80^th^ and 100^th^ amino acids. We also included the 42^nd^ and 53^rd^ amino acids because the insertion of a FP at these sites may improve membrane targeting of probes [13]. We tested a total of 39 different combinations of these insertion sites (Table 1). Each Nabi probe has a name composed of a version number 1 or 2, followed by a decimal point and an identification number (Table 1). All Nabi1 probes terminated after UKG except for Nabi1.243 which included the PTEN (phosphatase and tensin homolog) -like cytoplasmic domain of the phosphatase at the C-terminus (Table 1).

Nabi1 probes were evaluated by monitoring optical signals in response to 100 ms voltage steps in HEK293 cells using the voltage step protocols described in Materials and Methods and illustrated in Fig 1. Analyses of the signals from Nabi1 probes are given in Table 1. Fig 1C shows signal traces of single trials from 3 representative Nabi1 probes: Nabi1.213 (ΔF/F = 6%, -4% for acceptor and donor respectively for a 100 mV depolarization), Nabi1.242 (ΔF/F = 4%, -3%), and Nabi1.244 (ΔF/F = 2%, -2%). All three probes had both donor and acceptor signals with depolarizations while none had significant optical signals during hyperpolarizations. We also measured the signals from VSFP Butterfly1.2, a recently developed FRET based voltage sensor (Fig 1D) [13]. In contrast to the Nabi1 signals which are from single trials, the VSFP Butterlfy1.2 traces are the average of 16 trials. The VSFP Butterfly 1.2 signals are shown at two signal scales. The Nabi1 probes have a larger ΔF/F and a larger signal-to-noise ratio than VSFP Butterfly 1.2. The time course of the signals of both Nabi1 probes and VSFP Butterfly1.2 are better fit by double exponential functions than by single exponentials.

#### Signal size

**Fig 2:**
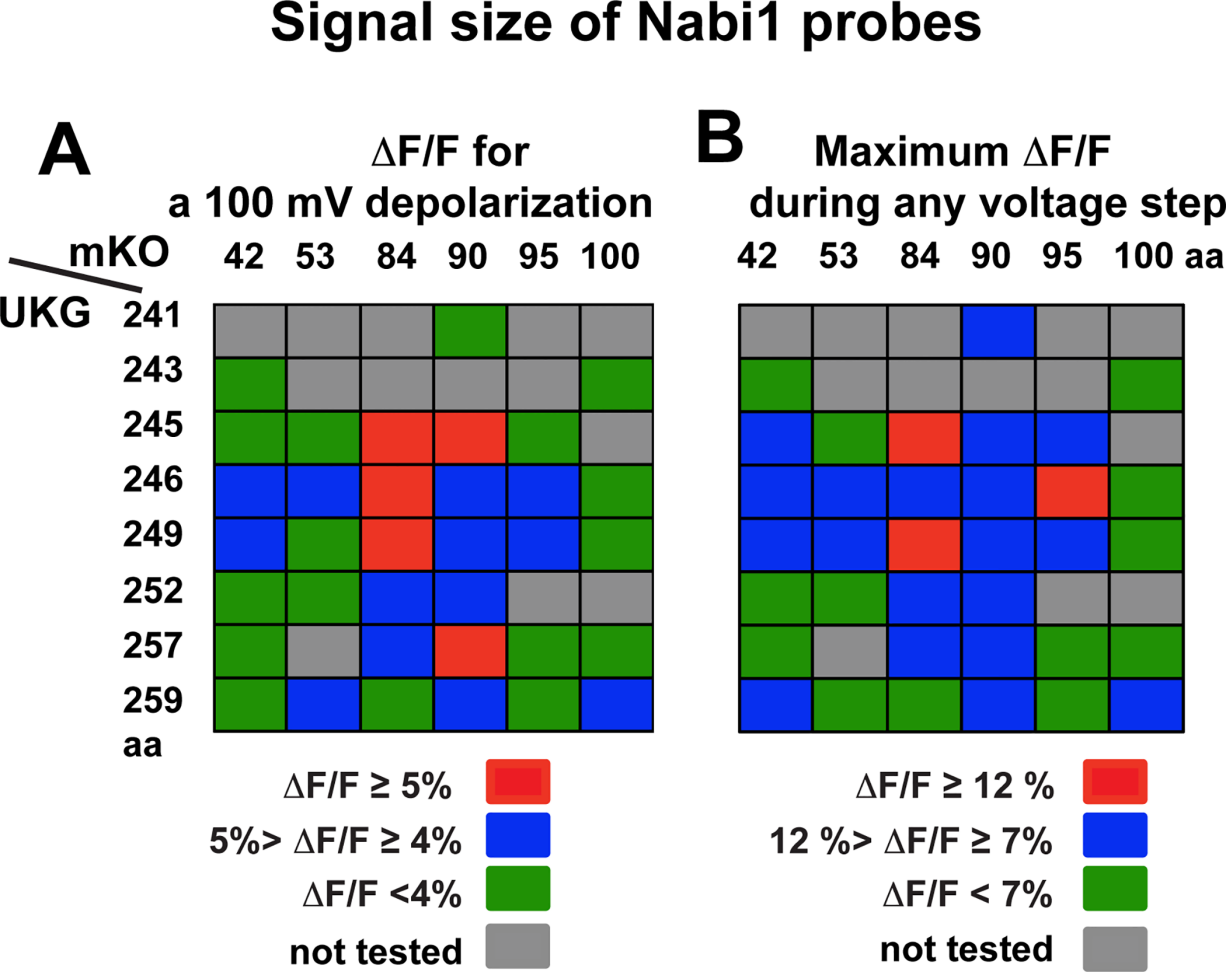
Signal sizes of Nabi1 probes in HEK293 cells. Positions of mKO and UKG are indicated. Grey rectangles indicate the combinations that were not tested. Nabi1 variants Nabi1.82, Nabi1.102, Nabi1.243, and Nabi1.152 (Table 1) are not included in this figure or Figs 3 and 4 (see Table 1). **A.** ΔF/F of Nabi1 optical responses for a 100 mV depolarization. ΔF/F was calculated by taking the averages of the optical responses for a 100 mv depolarization from 3–21 cells for each probe. Red rectangles indicate Nabi1 probes with ΔF/F of either donor or acceptor signal equal to or greater than 5%, blue rectangles indicate probes with ΔF/F between 4 and 5%, and green rectangles indicate probes with ΔF/F < 4%. Detailed ΔF/F values are listed in Table 1. **B.** Maximum ΔF/F during voltage steps between -170 mV and +130 mV from a -70 mV holding potential. Red rectangles indicate Nabi1 probes with ΔF/F_max_ of donor or acceptor signal equal to or greater than 12%, blue rectangles indicate probes with ΔF/F between 7 and 12%, and green rectangles indicate probes with ΔF/F < 7%. Detailed ΔF/F_max_ values are listed in Table 1.

The signal size (ΔF/F) for a 100 mV depolarization as a function of the locations of the FPs for Nabi1 probes are listed in Table 1 and illustrated schematically in Fig 2A. The locations of the FPs vs the largest signal size (ΔF/F_max_) observed during any voltage step from -70 mV are illustrated in Fig 2B. Clearly, the insertion sites of mKO and UKG in the cytoplasmic N- and C-termini influenced signal size. Half of tested Nabi1 probes showed ΔF/F equal to or higher than 4% for a 100 mV depolarization. While strong responders contained mKO at the 84^th^, 90^th^, or 95^th^ amino acid, located in the cytoplasmic segment near S1, and UKG between 245^th^ and 257^th^ amino acids, downstream from the S4, large FRET responses involved coordination of the two locations.

#### Signal kinetics

**Fig 3:**
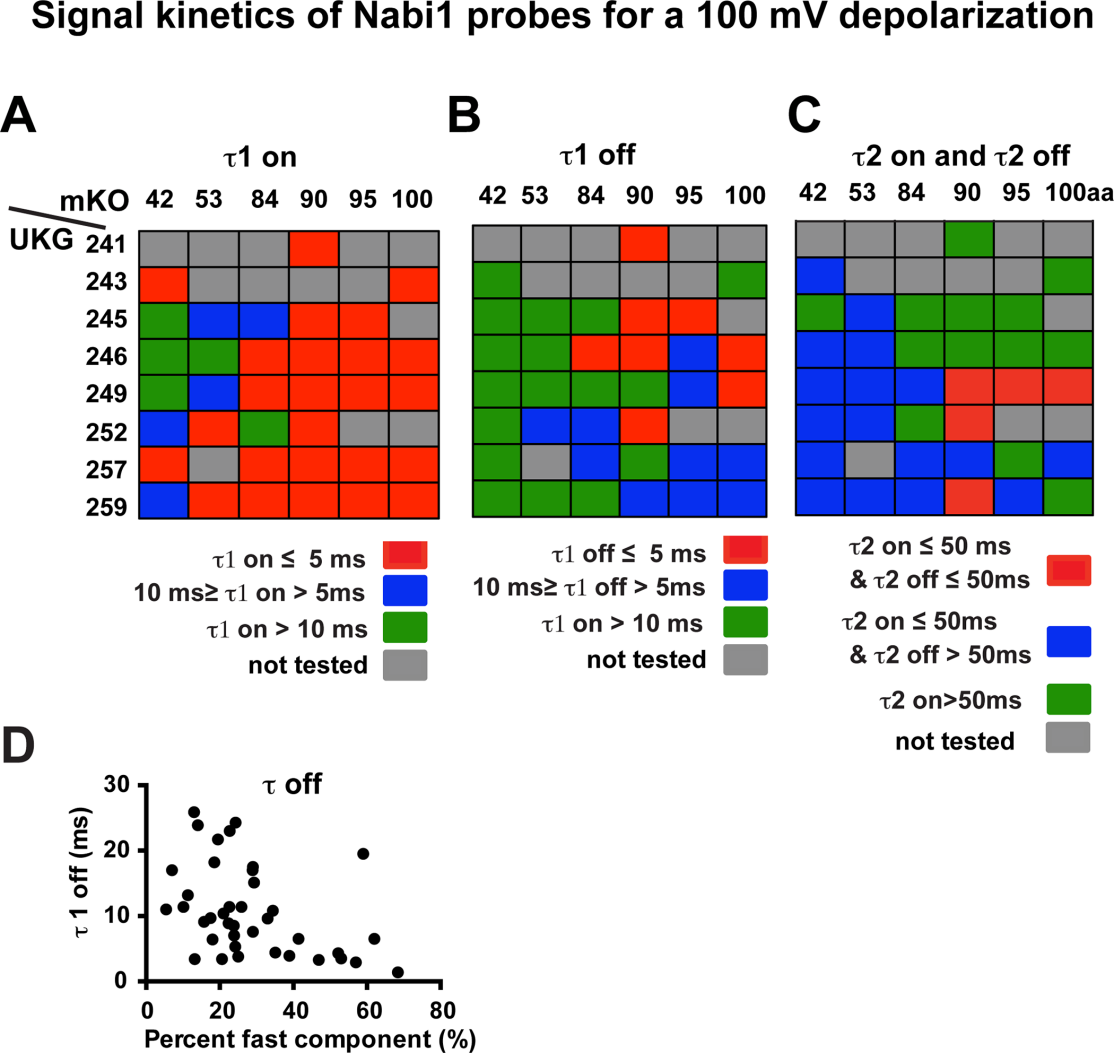
Time constants for signal activation and decay of the donor signal for Nabi1 probes in response to a 100 mV depolarization. Optical responses were analyzed using a double exponential function. Averages were taken from signals of 1–5 representative cells. Detailed values are listed in Table 1. Positions of mKO and UKG are indicated. Grey rectangles indicate the combinations that were not tested. **A.** Fast components of signal activation (τ1_on_). Red rectangles indicate probes with τ1_on_ faster than or equal to 5 ms. Blue rectangles indicate probes with τ1_on_ equal to or faster than 10 ms but slower than 5 ms. Green rectangles indicate probes with τ1_on_ slower than 10 ms. **B.** Fast components of signal recovery (τ1_off_). Same color scale as **A. C.** The summary of signal activation and recovery kinetics for the slow components of Nabi1. Red rectangles indicate probes with τ2 of signal activation faster than or equal to 50 ms and τ2 of signal decay equal to or faster than 50 ms. Blue rectangles indicate probes with τ2 of activation equal to or faster than 50 ms and τ2 of decay slower than 50 ms. Green rectangles indicate probes with τ2 of activation slower than 50 ms. **D.** The signal decay kinetics of Nabi1 probes showed an inverse relationship with percent fast components (R square = 0.17, p = 0.01).

The signal kinetics of the Nabi1 probes are presented in Table 1 and shown schematically as a function of the positions of the FPs in Fig 3. As suggested by the time courses of the Nabi1 signals in Fig 1, the response to 100 ms voltage steps were always best fit by two exponential components, labeled τ1 and τ2. Several Nabi1 probes had τ1 signals as fast as 2–3 ms (Table 1). While many Nabi1 probes had fast τ1 on kinetics (Fig 3A), only a few of them had fast τ1 off kinetics (Fig 3B). It appears that insertion locations closer to the transmembrane domain in the amino terminal cytoplasmic segment (mKO between the 84th or 100th amino acid) and insertion locations closer to the transmembrane domain in the carboxyl terminal cytoplasmic segment (UKG at the 241^st^ and 252^nd^ amino acid) give faster τ1-off values. A few of Nabi1 probes with insertion of mKO between the 84^th^ and 100^th^ amino acids and with UKG insertion between 241^st^ and 252^nd^ had fast signals for both activation and inactivation (Fig 3A and 3B). Again, signal kinetics appeared to be determined by coordination of two FP locations. For example, Nabi1.216 with mKO at the 84^th^ and UKG at the 246^th^ produced faster signal activation (τ1 on 2 ms) and faster signal decay (τ1 off 5 ms) compared to Nabi1.213 with mKO at the 84^th^ and UKG at the 245^th^ (τ1 on 7 ms; τ1 off 12 ms). (Fig 3A and 3B, Table 1). A few of Nabi1 probes with insertion of mKO between the 90^th^ and 100^th^ amino acids and UKG with insertion between 249^th^ and 259^th^ had τ2 signals that were relatively fast both for activation and inactivation (Fig 3C).

The signal activation of Nabi1 probes had 41 ± 2% fast component for a 100 mV depolarization during a 100 ms voltage step. The fraction of the fast component of signal activation was not strongly influenced by the positions of FPs (data not shown). The signal decay had 28 ± 3% fast component. Some probes with mKO at the 90^th^ or 95^th^ amino acid, and with UKG between the 240^th^ and 252^th^ amino acids exhibited a signal decay with more than 40% of fast component for a 100 mV depolarization (data not shown). The τ1 off value was inversely correlated with percent fast component (Fig 3D, R square = 0.17, p = 0.01), indicating that the probes with faster signal decays tended to have a larger fast component. Because the fraction of the fast component depends on the duration of the voltage step, these values are not included in Table 1.

We did not observe significant difference in the kinetics of the donor and acceptor signals of Nabi1 probes. For example, Nabi1.213, Nabi1.242, and Nabi1.244 displayed similar time constants for donor and acceptor signals during activation and decay (S1 Fig). Nabi1.213 and Nabi1.244 showed no difference in the fraction of fast component for donor and acceptor signals during activation and decay. The acceptor signal of Nabi1.242 had a significantly smaller fraction of fast component compared to the donor signal during activation (p = 0.01) while it showed no difference in percent fast component of acceptor and donor signals during signal decay.

#### V_1/2_ (the membrane potential at half maximum ΔF/F)

**Fig 4:**
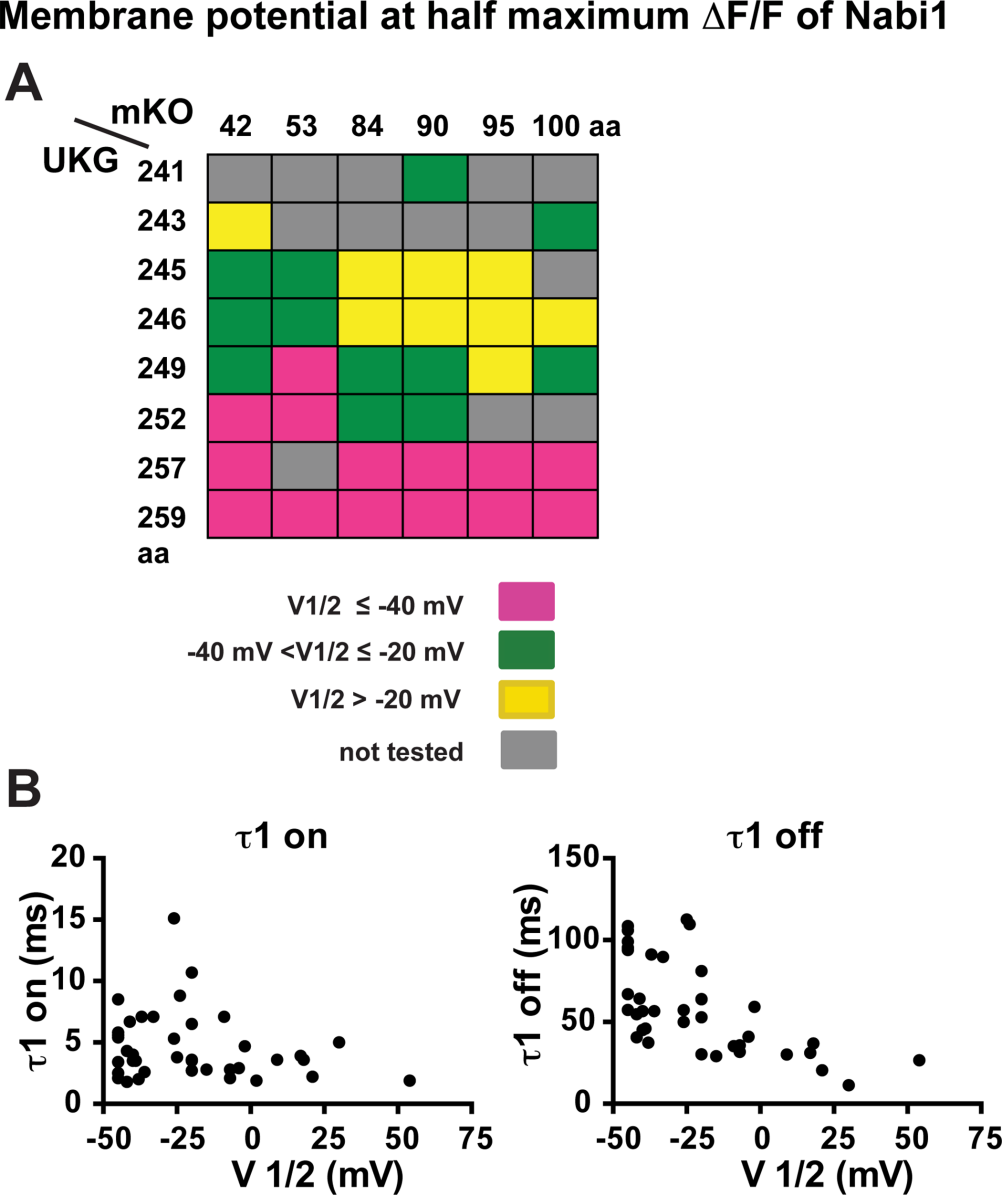
Membrane potential at half maximum ΔF/F (V_1/2_) of Nabi1 probes in HEK293 cells. **A.** V_1/2_ of Nabi1 probes. Positions of mKO and UKG are indicated. Grey rectangles indicate the combinations that were not tested. V_1/2_ was estimated from normalized donor ΔF/F of 2–14 measurements for each probe (see Materials and Methods). Detailed values are listed in Table 1. Purple squares indicate probes with V_1/2_ at more negative values than -40mV, green squares indicate probes with V_1/2_ between -40mV and -20mV, and yellow squares indicate probes with V_1/2_ that are more positive than -20 mV. **B.** The relationship between V_1/2_ and the time constants of optical responses. The left panel shows the relationship for τ1 of signal activation. There was no significant correlation (R square = 0.04, p = 0.22). The right panel indicates an inverse relationship with τ1 for signal decay. The Nabi1 probes with more negative V_1/2_ tend to have slower τ1 off values (R square = 0.38, P < 0.01).

All Nabi1 probes with V_1/2_ lower than -40 mV had UKG between the 249^th^ and the 259^th^ amino acids, downstream from the cytoplasmic end of S4 (Fig 4). In contrast, the positions of mKO at the N-terminus appeared to have only a marginal influence. As expected, the probes with low V_1/2_ displayed relatively large hyperpolarizing responses (ΔF/F >2%) compared to other Nabi1 probes (data not shown). Interestingly, Nabi1 probes with a more hyperpolarized V_1/2_ had slower signal decay τ1 kinetics (Fig 4B right; R square = 0.38, p < 0.01) while they did not exhibit a significant relationship with τ1 on (Fig 4B left; R square = 0.04, p = 0.22).

#### Nabi1 variants

Nabi1.243 was identical to Nabi1.242 except that it has the PTEN-like C-terminal phosphatase domain following UKG. The V_1/2_ for Nabi1.243 was shifted toward a more positive potential (+16 mV for Nabi1.242; +53 mV for Nabi1.243). The maximum signal size for the two sensors was similar (Table 1). In addition to Nabi1.243, Nabi1.82, Nabi 1.102, and Nabi1.152 (Table 1) had unusual amino acid sequences (see Table 1 legend) and the data from them are not included in the analyses of Figs 2, 3 or 4.

#### Neuron expression of Nabi1 sensors

Based on signal size and kinetics, we chose several Nabi1 probes including Nabi1.213, Nabi1.242, and Nabi1.244, and transiently expressed them in primary cultured hippocampal neurons. However, the expression of these proteins mainly resulted in intracellular aggregates (data not shown), consistent with a previous report that Mermaid, which also contained the FPs mKO and UKG, expressed poorly in primary hippocampal neurons [21]. We were not able to detect action potential signals from Nabi1 in neurons and decided to modify several Nabi1 constructs by replacing the FRET pair.

### Nabi2 FP voltage sensors

**Fig 5:**
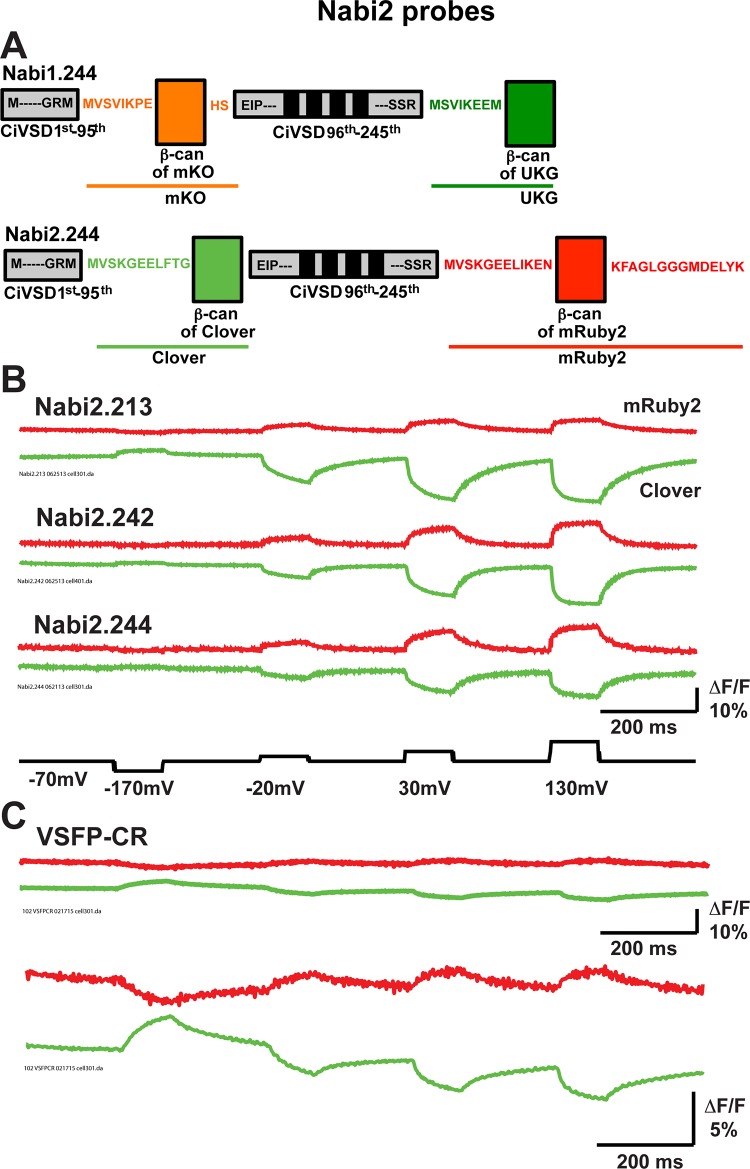
Nabi2 probes. **A.** Nabi2 has Clover (green) and mRuby2 (red) as the FRET pair. Nabi1 and Nabi2 are also different in the linkers between the *Ciona* voltage sensing domain (CiVSD) and FP β-cans. The *Ciona* voltage sensitive domain and the N-terminus are shown in gray. The four transmembrane domains S1-S4 are indicated as black bars. FPs are color-coded as mKO (orange), UKG (dark green), Clover (bright green), and mRuby2 (red). β-cans of the FPs are shown as vertical rectangles. **B.** Representative donor (Clover; green) and acceptor (mRuby2; red) signals of Nabi2.213, Nabi2.242, and Nabi2.244. All of the traces were from single trials and without temporal filtering. **C.** Our results using VSFP-CR [20] are shown from single trials using scale bars of ΔF/F of 10% and 5% and without temporal filtering for comparison. The same voltage steps as in **B** were used for VSFP-CR.

The Nabi2 constructs were generated by replacing the FRET pair of Nabi1 with Clover (green) and mRuby2 (red) (Fig 5A) [20]. The Clover/mRuby2 pair of VSFP-CR was previously shown to be able to substitute for the FRET pair of VSFP2.3 [21] in transiently expressed neurons [20]. We converted eight Nabi1 sensors into Nabi2 sensors (Table 2) by replacing Clover at the position for mKO and mRuby2 at the position for UKG (Fig 5A). We selected the eight based on signal size and kinetics (Figs 2 and 3 and Table 1). The Nabi2 sensors have the donor FP at the N-terminus in contrast to Nabi1 (see rationale in Materials and Methods). Replacement of FPs also resulted in changes to the linkers between the *Ciona* voltage sensing domain and the β-can structures of the FPs (Fig 5A). Signal traces from single trials of Nabi2.213, Nabi2.242, and Nabi2.244 are shown in Fig 5B. The signal-to-noise ratios of the Nabi2 responses in HEK293 cells tended to be better than those for the Nabi1 analogues (compare Figs 1C and 5B). In our hands, the signal sizes and on/off kinetics of Nabi2 sensors were better than VSFP-CR (Fig 5B and 5C). Voltage sensors with Clover (donor FP) alone produced only negligible optical signals in the absence of mRuby2 (acceptor FP) in the same construct (M. Shepheri-Rad and L. Cohen, unpublished observation). Similar observations were reported with Mermaid 2; neither CFP alone nor YFP alone had a substantial optical signal [8]. After data acquisition for 4 trials lasting 1.6 sec under arc lamp illumination as described in Materials and Methods, the fluorescent intensity of donor was reduced to 83 ± 2% for Nabi2.242 (N = 6) and 83 ±1% for Nabi2.244 (N = 6) compared to the intensity before the trials, and the fluorescent intensity of acceptor was reduced to 86 ±1% for Nabi2.242 (N = 6) and 84 ± 2% for Nabi2.244 (N = 6). We compared donor signal traces of Nabi2.242 collected from the outline and from the entire cell (S2 Fig). Collecting signals from the outline of the cell (plasma membrane) did not greatly improve the signal-to-noise ratio in comparison to the signals collected from the entire cell.

**Table 2:** Comparison of Nabi1 and Nabi2 probes. **Δ**F/F, time constants, and V_1/2_ were compared using averaged optical traces of 16 trials in transiently transfected HEK293 cells. ΔF/F and time constants were evaluated from optical responses of the donor to a 100 mV depolarization. We examined 5–20 cells for each probe. V_1/2_ was evaluated by fitting with a Boltzmann function.

Nabi probes (locations of FPs)	ΔF/F	τ1 on	τ2 on	Fast component of on	τ1 off	τ2 off	Fast component of off	V_1/2_ of donor	V_1/2_ of acceptor
	%	ms	ms	%	ms	ms	%	mV	mV
Nabi1.213 (84^th^, 245^th^)	-6±1	4 ±1	42± 7	31 ± 2	11± 2	50± 5	38± 6	-24± 3	-12± 3
Nabi2.213 (84^th^, 245^th^)	- 6 ±1	3 ± 0	39 ± 5	35 ± 3	9 ± 1	68± 4	21 ± 1	-31 ± 3	-33 ± 3
Nabi1.216 (84^th^, 246^th^)	-5 ± 1	3 ± 0	49 ± 6	34 ± 4	4 ± 1	52 ± 7	10 ± 2	-30 ± 3	-32 ± 5
Nabi2.216 (84^th^, 246^th^)	-5 ± 1	1 ± 0	17 ± 3	20 ± 4	2 ± 1	30 ± 4	5 ± 1	-58 ± 5	-65 ± 4
Nabi1.30 (90^th^, 241^st^)	-3 ± 1	3 ± 1	55± 13	36 ± 9	5 ± 1	69 ± 15	24 ± 7	-66± 11	-43 ± 4
Nabi2.30 (90^th^, 241^st^)	-4 ± 1	3 ± 1	34 ± 8	28 ± 9	4 ± 2	45 ± 6	19 ± 2	-70 ± 7	-73 ± 11
Nabi1.226 (90^th^, 245^th^)	-5 ± 1	3 ± 0	72± 19	36 ± 6	3 ± 1	35 ± 4	22 ± 11	-15 ± 8	-37 ± 16
Nabi2.226 (90^th^, 245^th^)	-5 ± 1	2 ± 1	21 ± 0	40 ± 3	3 ± 1	40 ± 7	14 ± 2	-65 ± 2	-83 ± 12
Nabi1.125 (90^th^, 246^th^)	-3 ± 1	2 ± 0	45 ± 9	43 ± 5	4 ± 1	52 ± 9	28 ± 5	-26 ± 9	-16 ± 9
Nabi2.125 (90^th^, 246^th^)	-4 ± 1	2 ± 0	17 ± 3	35 ± 6	2 ± 0	39 ± 7	10 ± 2	-51 ± 7	-66 ± 13
Nabi1.104 (90^th^, 252^nd^)	-3 ± 0	2 ± 0	32 ± 6	38 ± 3	3 ± 2	39 ± 4	11 ± 6	-54 ± 5	-39 ± 0
Nabi2.104 (90^th^, 252^nd^)	-4 ± 1	2 ± 0	19 ± 2	35 ± 4	3 ± 1	41 ± 2	15 ± 5	-66 ± 3	-72 ± 4
Nabi1.244 (95^th^, 245^th^)	-2 ± 0	8 ± 3	85± 19	23 ± 7	6 ± 1	60± 9	26 ± 6	57 ± 12	59 ± 9
Nabi2.244 (95^th^, 245^th^)	-5 ± 1	6 ± 2	65 ± 9	34 ± 3	7± 1	42 ± 2	25 ± 3	-12 ± 6	-4 ± 5
Nabi1.242 (95^th^, 246^th^)	-4 ±1	10±2	73±14	42 ± 2	14± 3	60±10	52 ± 7	46 ± 5	47± 2
Nabi2.242 (95^th^, 246^th^)	-5 ± 1	5 ± 0	59± 13	39 ± 1	7 ± 1	51 ± 6	27 ± 4	**-**13 ± 1	-6 ± 3

#### Signal size and kinetics

**Fig 6:**
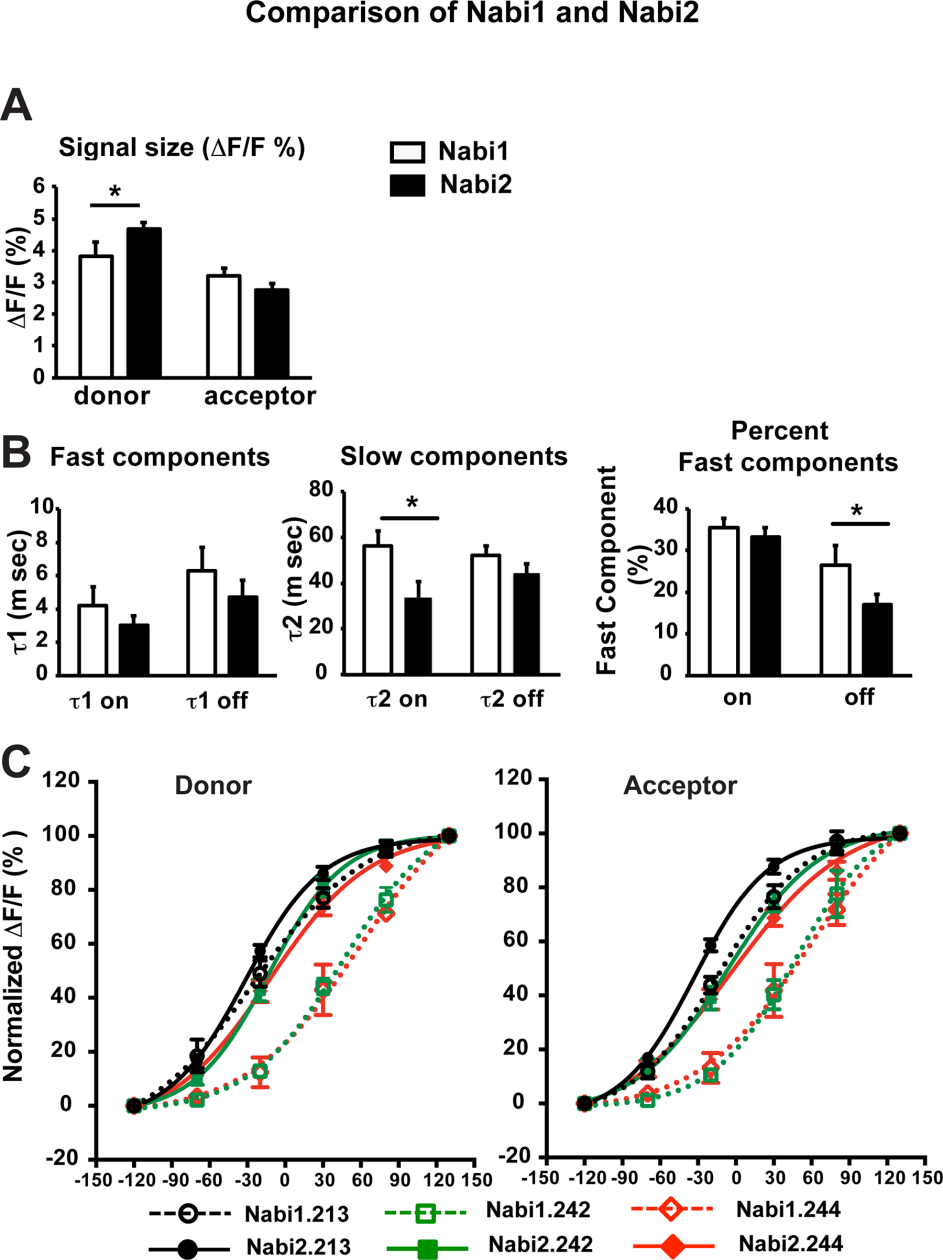
Comparison of Nabi1 and Nabi2 probes. **A-D**. Signal size and time constants of eight Nabi1 and eight Nabi2 probes were averaged and compared by a t test. An asterisk (*) indicates statistical differences (p<0.05). **A.** ΔF/F of Nabi1 and Nabi2 probes for a 100 mV depolarization in HEK cells. The signals were taken from the averaged signals of Nabi1 or Nabi2 probes. Converting of Nabi1 to Nabi2 probes increased donor signal size, but not acceptor’s. **B.** Comparison of the time constants of the optical responses of donor to a 100 mV depolarization. Left: τ1 of signal activation and decay. Nabi1 and Nabi2 were not significantly different. Middle: Nabi2 had faster slow component of signal activation compared to Nabi1 (p = 0.03). Signal decay did not change significantly. Right: Replacement of FPs did not change significantly the percent fast component of signal activation, but reduced the percent fast component of signal decay in Nabi2 compared to Nabi1 (p = 0.03). **C.** Signal vs. voltage for three Nabi1 and Nabi2 probes. ΔF/F values were normalized to the maximum ΔF/F of each Nabi probe (N = 6–8 cells for Nabi1 probes, 14–19 cells for Nabi2 probes) and fit by a Boltzmann function. Left panel: donor signals *vs* voltage. Right panel: acceptor signals vs voltage. Dotted lines with open symbols are for Nabi1 probes and solid lines with closed symbols are for Nabi2 probes.

We further compared the eight Nabi1 and eight Nabi2 probes in Fig 6 and Table 2. Replacing FPs resulted in some increase of donor signal size (ΔF/F), but did not change signal size for acceptor (Fig 6A). Like Nabi1, the time courses of the Nabi2 signals were better fit by double exponential functions (S3 Fig). The signal kinetics of Nabi2 probes were similar to Nabi1 (Fig 6B) except some improvement of the slow component of signal activation (Fig 6B middle) and a reduction of the fraction of fast component of signal decay (Fig 6B right). Nabi2.213, Nabi2.242, and Nabi2.244 displayed similar time constants and percent fast components for acceptor and donor signals during activation and decay (S1 Fig).

#### V_1/2_

The signal (ΔF/F) vs voltage curves of both the donor and acceptor fluorescence of the Nabi1 and Nabi2 probes had sigmoidal relationships and were fit by a Boltzmann equation (Fig 6C, Table 2). Signal vs voltage curves for Nabi1&2.213, Nabi1&2.242, and Nabi1&2.242 are shown in Fig 6C. Each of the Nabi2 probes responded at more negative membrane potential compared to the corresponding Nabi1 probe for both donor and acceptor signals (Fig 6, Table 2). The V_1/2_ of the eight donor signals of Nabi2 sensors were shifted by -31.8 ± 8.8 mV from signals of Nabi1 probes (p < 0.01 by a t test) and acceptor signals of Nabi 2 probes were shifted V_1/2_ by -41.3 ± 4.9 mV (p < 0.01 by a t test; Table 2). The changes in V_1/2_ values of donor and acceptor signals were not significantly different (p = 0.36 by a t test).

#### Neuron signals

**Fig 7:**
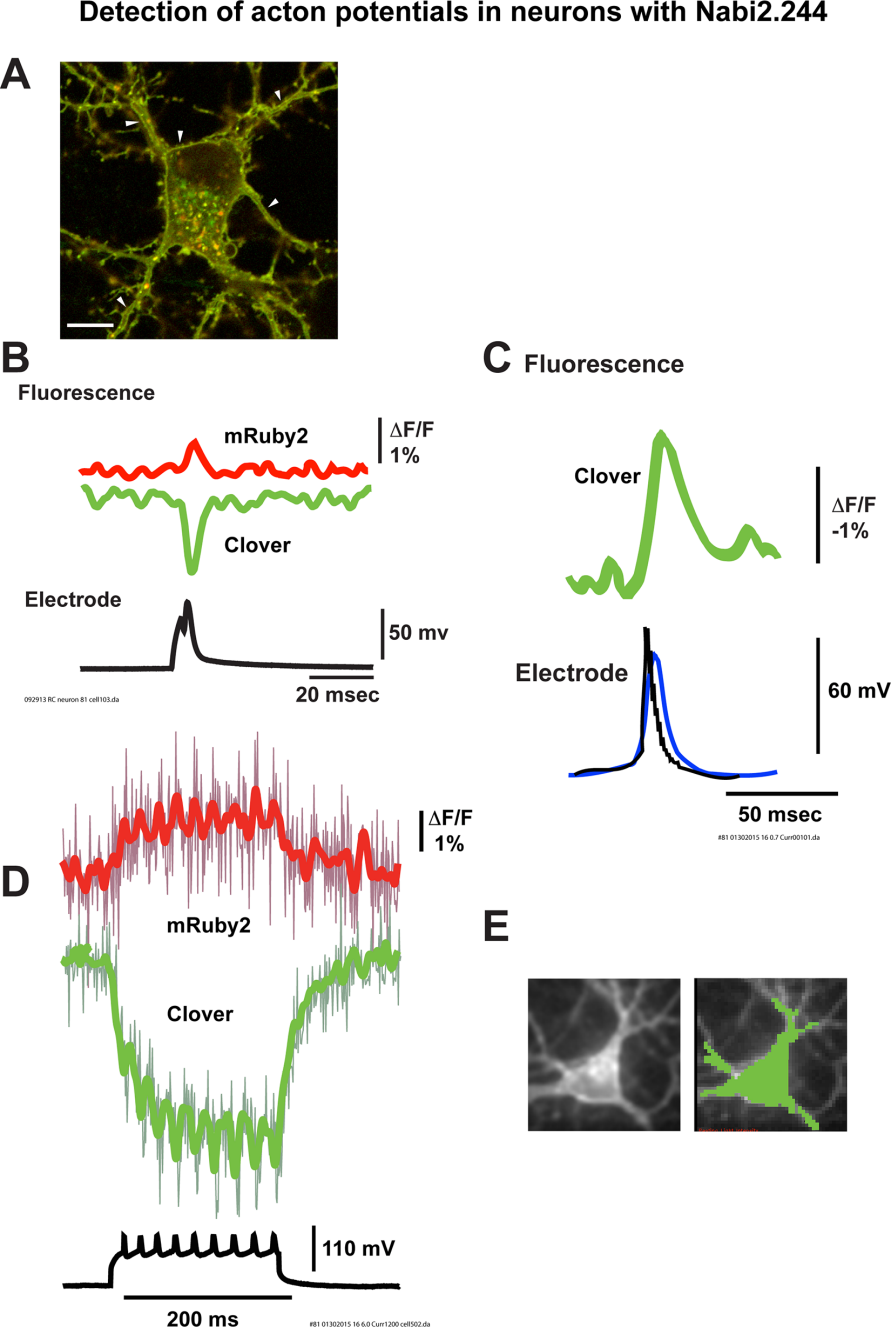
Nabi2.244 responded to action potentials in primary cultured hippocampal neurons. **A.** Expression of Nabi2.244 in a neuron, as shown with confocal laser scanning microscopy. A merged image of clover/mRuby2 is shown. The scale bar indicates 10 μm. Nabi2.244 was partially localized in the plasma membrane of soma and processes. Arrowheads point to membrane localization of fluorescent signals. Nabi2.244 was also found in intracellular aggregates. **B.** Representative donor (green) and acceptor (red) signal traces from a single trial (Fluorescence) responding to a stimulated action potential (Electrode) from a neuron expressing Nabi2.244. Signal traces were smoothed by two passes of a low pass binomial filter. **C.** A representative donor signal from a single trial (Fluorescence; green) responding to a spontaneous action potential (Electrode; black trace is without filtering and blue trace is with filtering) from a neuron expressing Nabi2.244. A single signal trace is shown smoothed by a low pass Gaussian filter at 50 Hz. **D.** Representative donor (green) and acceptor (red) signal traces from a single trial during a train of stimulated action potentials (black) from a neuron expressing Nabi2.244. Optical traces from a single trial of a current clamped cell are shown smoothed by 10 passes of a low pass binomial filter. Unfiltered signal traces (purple and dark green) are superimposed. **E.** An image of a neuron with resting fluorescence intensity (left). The optical signals in **D** were collected from the soma and the proximal processes, marked with green (right).

Nabi2.213, Nabi2.242 and Nabi2.244 all exhibited good plasma membrane localization in acutely cultured neuronal soma and processes with some cytoplasmic aggregates (Fig 7A for Nabi2.244). All 3 probes had measurable responses to single action potentials; Single trial results for Nabi2.244 are shown in Fig 7. Signals from donor and acceptor responding to an evoked individual action potential were clearly detectable (Fig 7B). As was the case in HEK293 cells (Fig 5B), the donor signals were larger than the acceptor signals. The donor signal of Nabi2.244 had a fractional fluorescence change of 1.7 ± 0.1% (N = 6). The full width at half maximum of these optical signals was 3.3 ± 0.2 ms for the donor (N = 16) and 3.5 ± 0.3 ms for the acceptor (N = 7). Nabi2.213 and Nabi2.242 had similar optical responses to evoked action potentials (data not shown). Nabi2.244 responded to a spontaneous action potential, as shown with the donor signal from a single trial (Fig 7C). Nabi2.244 was further evaluated for the responses to a train of action potentials elicited by a current clamp step in hippocampal neurons. Optical responses of Nabi2.244 to spikes at 45 Hz were observed both for donor and acceptor (Fig 7D). The signals were the average of all the pixels receiving light from the soma and the proximal part of processes indicated by green color in the right panel of Fig 7E.

#### The tau1 contribution for shorter voltage steps

Because the 100 ms voltage steps we used with the HEK293 cells are much longer than the duration of an action potential, we also estimated the amplitude contribution of the two components to a 5 ms voltage step. The values for the Nabi2 probes are shown in Table 3. The fast component of the fluorescence change accounted for most of the responses to a 5 ms step.

**Table 3:** Calculated responses of Nabi2 probes for a 5 ms 100 mV depolarization. Averaged donor traces of 16 trials were obtained from transiently transfected HEK293 cells, fit by a double exponential function, and used to calculate the responses at 5 ms.

	ΔF/F	Contribution of fast component
	%	%
Nabi2.213	-2.3 ± 0.6	78 ± 3
Nabi2.242	-3.2 ± 0.5	65 ± 3
Nabi2.244	-3.5 ± 0.8	65 ± 10

## Discussion

### Locations of the fluorescent proteins and the voltage responses

#### Signal size and kinetics

The positions of the fluorescent donor and acceptor in the Nabi1 proteins affected the signal size and the response time constants. Many Nabi1 probes have a large signal amplitude (up to 17% ΔF/F_max_) and relatively fast signal kinetics (Figs 1–3, Table 1). Large fluorescent signals and fast responses came from coordination of the locations of donor and acceptor. The hot spots for large signal size and fast kinetics were largely clustered between the 84^th^ and 100^th^ amino acids for mKO and between the 241^st^ and 257^th^ amino acids for UKG (Figs 2 and 3). Several Nabi1 probes such as Nabi1.216, Nabi1.30, Nabi1.226, Nabi1.125, Nabi1.104, Nabi1.244, and Nabi1.49 exhibited fast signal kinetics for both activation (τ1 on = ~2 ms) and decay (τ1 off = ~3 ms) (Fig 3, Table 1). We report the τ values for a 100 mv depolarization, but it is important to note that the τ values of the signals are voltage dependent; generally faster τ values for larger depolarizations (e.g., Figs 1 and 5).

#### Comparison with other FRET probes

VSFP butterfly1.2 [13] and Mermaid2 [8] have similar “butterfly” structures to Nabi probes (the FRET FPs are flanking the S1-S4 transmembrane domain). VSFP Butterfly1.2 had mKate at the 69^th^ and mCitrine at the 250^th^ amino acids. Many Nabi1 probes with UKG insertion between 249^th^ and 259^th^ amino acids responded with fast signal activation but slow signal decay kinetics (Fig 3B, Table 1). Many Nabi1 and Nabi2 probes exhibited signals with larger signal dynamics than VSFP Butterfly1.2 [13] and responded with faster on and off kinetics (Fig 1C and 1D). Mermaid2 and Mermaid2β [8] had mUKG or CFP at the 103^th^ and mKO or YFP at the 249^th^ amino acids. Mermaid 2 showed a signal size up to 20%, comparable to signal sizes of Nabi1 (Table 1), in HEK293 cells [8]. Mermaid2 exhibited fast on-kinetics, but had relatively slow decay kinetics [8]. Signals of Nabi2 probes were larger and faster than VSFP-CR (Fig 5B and 5C). VSFP-CR [20] has Clover and mRuby2 in tandem at the C-terminus of the voltage sensing domain with the same backbone of VSFP2.3 [21]. We conclude that by screening more FP locations, we have developed improved FRET based voltage sensors.

#### V_1/2_

The V_1/2_ was mainly determined by the position of UKG at the cytoplasmic C-terminus of the voltage sensitive domain, and marginally influenced by the position of mKO at the N-terminus (Fig 4). Interestingly, the Nabi1 probes with a low V_1/2_ had relatively slow signal decays (Fig 4B). We do not understand the reason for the relationship of V_1/2_ and UKG location or the relationship between V_1/2_ and signal decay kinetics.

### Effect of the two different fluorescent protein pairs on the voltage responses

#### Signal size and kinetics

The Nabi2 probes with Clover and mRuby2 had much better expression in neurons compared to Nabi1 probes with mKO and UKG. Changing the FPs did not alter the fast components of signal activation and decay (Fig 6B). Replacing the Nabi1 FRET pair with Clover/mRuby2 slowed down signal decay by reducing percent fast component (Fig 6, Table 2). Overall, we observed marginal, not dramatic, changes in signal kinetics by converting the Nabi1 probes into Nabi2.

#### V_1/2_

The fluorescence signal vs voltage relationship of all eight Nabi2 probes was shifted toward more negative potentials in comparison with the Nabi1 probes (Fig 6E, Table 2). Thus the signal-vs-voltage dependence appears to be influenced not only by locations of FPs in the backbone structure but also by the choice of the FRET pair. The amino acid sequences of the FPs UKG/mKO and Clover/mRuby2 are quite divergent (27% identity and 45% homology between UKG and Clover; 44% identity and 61% homology between mKO and mRuby2). One hypothesis for explaining the FRET signal is that it results from a voltage dependent shift in a monomer-dimer equilibrium between the two FPs. The shift of V_1/2_ with the change of FPs could arise from a difference in the monomer-dimer equilibrium between the two FP pairs [25–27]. However, in addition to this difference between FPs, replacing FP pairs resulted in changes in the linkers between the *Ciona* voltage sensing domain and the β-can structures of the FPs (Fig 5A). There were changes in both the lengths and amino acid compositions of the linkers. This difference in linkers could also underlie some of the functional differences between Nabi1 and Nabi2 probes, consistent with a recent report [28].

### Nabi probes as voltage sensors

ArcLight is known to produce a robust voltage dependent signal in cultured mammalian neurons [5] and in the *in vivo* fly [29]. Recently, ArcLight signals were measured in response to odor inhalation in the *in vivo* mouse olfactory bulb. A large fractional change (~4%) with a good signal-to-noise ratio was obtained in single trials [6]. The recently developed FP voltage sensor Bongwoori produced optical responses to action potentials at 60 Hz [9]. Nabi2 demonstrated signal kinetics equivalent to Bongwoori in neurons (Fig 7D). Although the signal size of Nabi2 probes was not as large as ArcLight in HEK293 cells [5], the faster kinetics of Nabi2 probes produced optical signals during action potentials that were comparable to those from ArcLight. The faster kinetics and reasonable signal sizes may make Nabi2 probes useful for *in vivo* recording. Nabi2 probes are fast voltage probes that can be useful for monitoring neuronal activity especially when being used with ratiometric imaging.

The locations of FPs for large optical signals and locations for fast responses tended to be clustered around amino acids closer to the S1-S4 domain (Figs 2 and 3). On the other hand, probes with low V_1/2_ had insertion of FPs distal to the S4 domain (Fig 4). In order to selectively monitor action potentials, voltage probes should respond to membrane potential above threshold with a large signal and fast kinetics. On the other hand, voltage probes with the ability to signal membrane potential changes near the resting potential may be useful for detection of sub threshold synaptic potentials. Nabi2 exhibited left-shifted signal *vs* voltage responses compared to Nabi1 (Fig 5E). Some Nabi2 probes are more responsive to action potentials while others will be relatively selective for membrane potentials around the resting potential.

Expression of FP-voltage sensors may add an extra capacitance to the membrane and influence the spiking properties of the target cells. ArcLight expression in cultured neurons did not affect the time course and amplitude of action potentials [5] and photobleaching and phototoxicity were not detected in *in vivo* measurements. The effect of capacitative load associated with VSFP2.3 (a FRET-based probe) and VSFP3.1 (a single FP probe) have been estimated by computational modeling [30]. The use of the *Ciona* voltage sensitive domain where the FP to S4 ratio is 1:1 appears to result in a reduced capacitative load compared to the use of potassium channel based voltage probes.

Recent publications include new approaches for developing better fluorescent protein voltage sensors [2–4, 8–12]. Nabi2 probes have the largest signal sizes and fastest optical responses in neurons among the FRET based probes. It is not yet clear which of these types of probes will be most useful for *in vivo* measurements of mammalian brain activity or which approach will lead to the most rapid further improvements. Considering brightness, photostability, pH sensitivity, and pharmacological and photodynamic effects [20], Nabi2 probes may well prove useful for imaging neuronal membrane potential.

We investigated the relationship of locations of the FPs and signal size and dynamics of “butterfly” type of FRET-based voltage sensors. We report Nabi2 probes with fast signal kinetics and optical responses large enough to distinguish individual action potentials in neuronal culture. The information about the relationship of locations of the FPs and probe function may provide strategic guidance for designing future voltage sensors.

## Supporting Information

S1 Fig Comparison of the donor and acceptor kinetics of Nabi1 and Nabi2 probes. Time constants of signal activation (**A, B**) and decay (**C, D**) kinetics, percent fast component of signal activation (**E, F**) and decay (**G, H**) of acceptor and donor signals for Nabi1 (**A, C, E, G**) and Nabi2 (**B, D, F, H**) probes are compared. The percent fast component of the acceptor signal of Nabi1.242 is slightly smaller than that of donor during signal activation (p = 0.01 by a t test). Otherwise, none of Nabi1 and Nabi2 probes displayed noticeable difference in signal kinetics of donor and acceptor.
(TIF)

S2 Fig Effect of ROI on signal size. The signals from the edge of the cells were ~10% larger than the average of all of the pixels. **A**. Left: image of an HEK293 cell taken with the 80x80 pixel NeuroCCD-SMQ camera used for fast imaging. Middle: Image of the cell overlaid with the pixels (green) used for the measurement of the edge signal. Right: Image of the cell overlaid with the pixels (red) used for the measurement of the average of all of the pixels receiving light from the cell. **B**. Donor traces showing the average of the edge (green) and all (red) pixels from a HEK293 cell expressing Nabi2.242. The traces are from a single trial; from the same data as shown in Fig 5B. The data was low pass filtered with two passes of a binomial 1-2-1 filter.
(TIF)

S3 Fig The evaluation of fit for the time course of fluorescence signals. Representative optical traces of Nabi2.213, Nabi2.242, and Nabi2.244 responding to a 100 mV depolarization were fit by double or single exponential functions to determine time constants for signal activation (on) or signal decay (off). Residuals to assess the quality of the fits are shown for each data point versus time.
(TIF)

S1 File ARRIVE Checklist. (PDF)
