# Supplementary figures and images for: Developing Fast Fluorescent Protein Voltage Sensors by Optimizing FRET Interactions
Source: PLoS One. 2015 Nov 20;10(11):e0141585. doi: 10.1371/journal.pone.0141585 (PMC4654489; doi:10.1371/journal.pone.0141585)

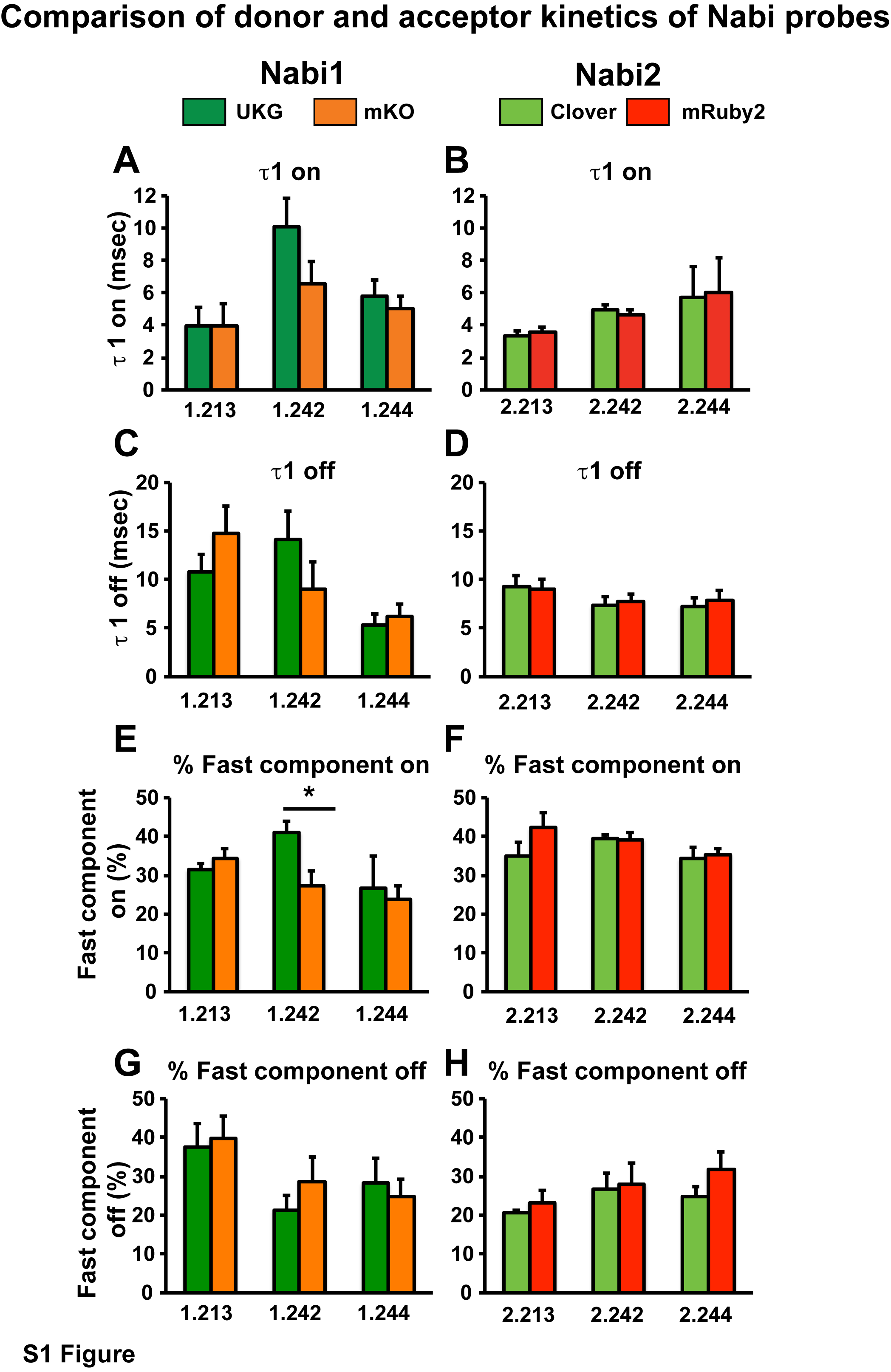

Supplement: S1 Fig — Time constants of signal activation (A, B) and decay (C, D) kinetics, percent fast component of signal activation (E, F) and decay (G, H) of acceptor and donor signals for Nabi1 (A, C, E, G) and Nabi2 (B, D, F, H) probes are compared. The percent fast component of the acceptor signal of Nabi1.242 is slightly smaller than that of donor during signal activation (p = 0.01 by a t test). Otherwise, none of Nabi1 and Nabi2 probes displayed noticeable difference in signal kinetics of donor and acceptor. (TIF) [file pone.0141585.s001.tif]

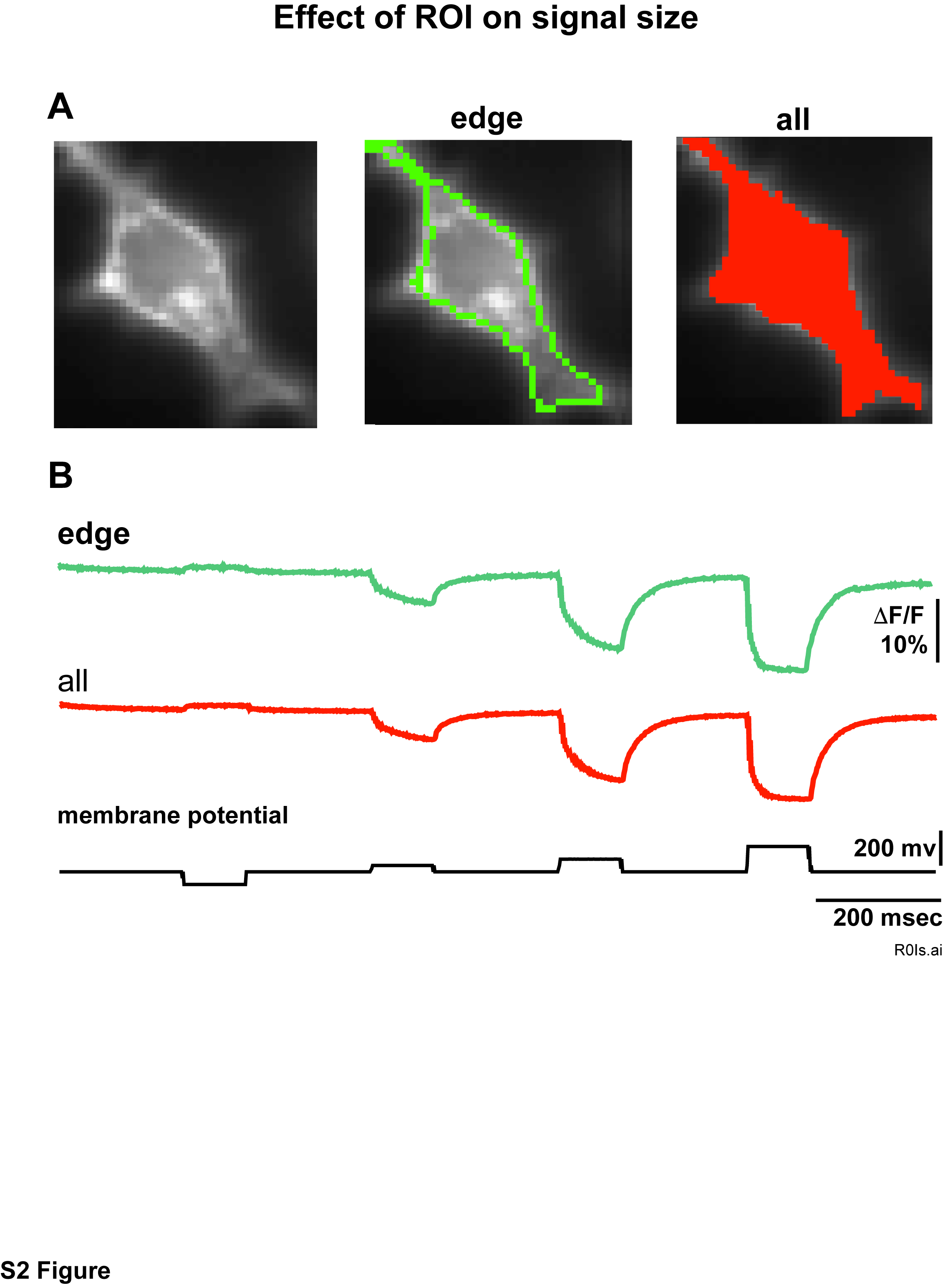

Supplement: S2 Fig — The signals from the edge of the cells were ~10% larger than the average of all of the pixels. A. Left: image of an HEK293 cell taken with the 80x80 pixel NeuroCCD-SMQ camera used for fast imaging. Middle: Image of the cell overlaid with the pixels (green) used for the measurement of the edge signal. Right: Image of the cell overlaid with the pixels (red) used for the measurement of the average of all of the pixels receiving light from the cell. B. Donor traces showing the average of the edge (green) and all (red) pixels from a HEK293 cell expressing Nabi2.242. The traces are from a single trial; from the same data as shown in Fig 5B. The data was low pass filtered with two passes of a binomial 1-2-1 filter. (TIF) [file pone.0141585.s002.tif]

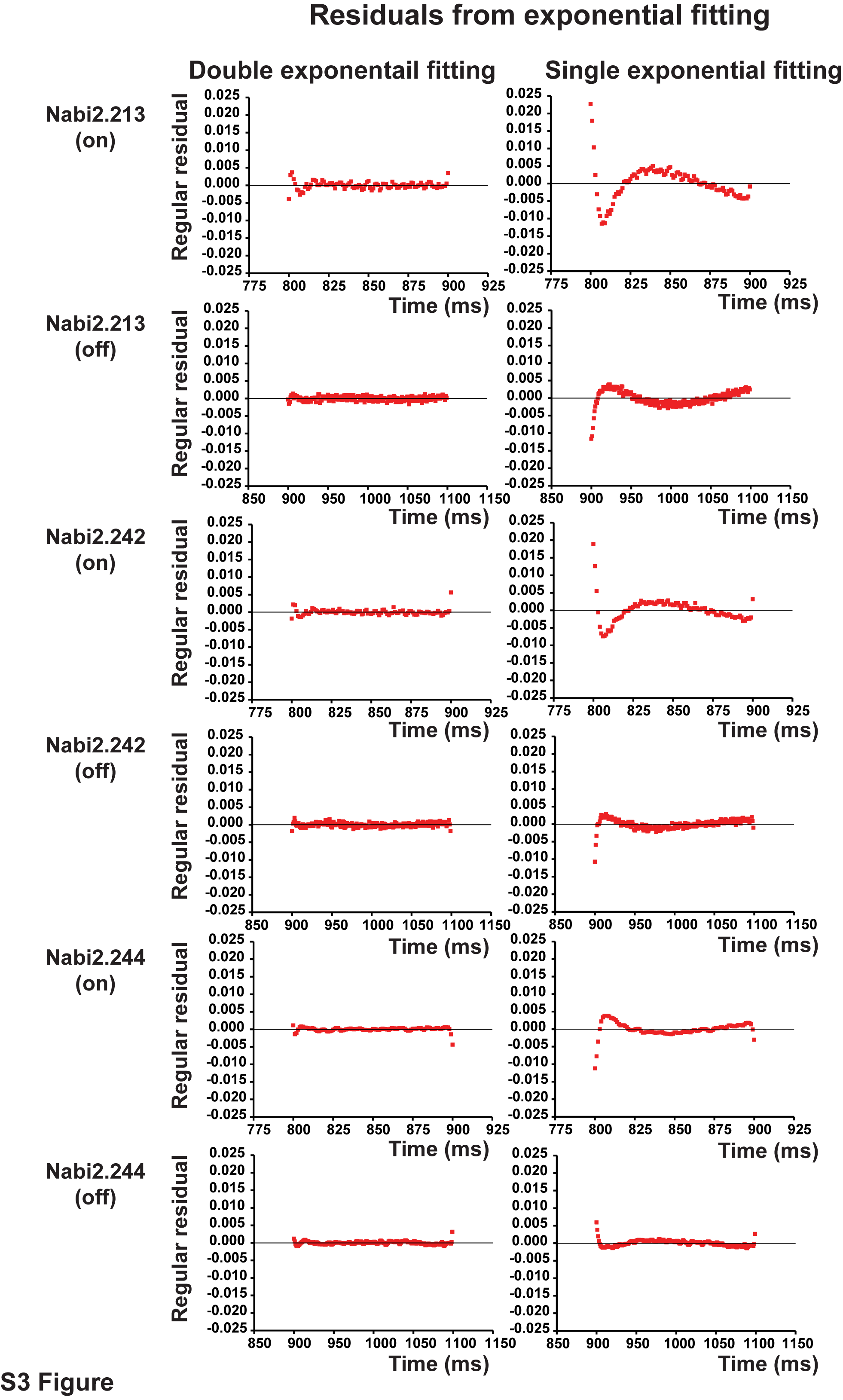

Supplement: S3 Fig — Representative optical traces of Nabi2.213, Nabi2.242, and Nabi2.244 responding to a 100 mV depolarization were fit by double or single exponential functions to determine time constants for signal activation (on) or signal decay (off). Residuals to assess the quality of the fits are shown for each data point versus time. (TIF) [file pone.0141585.s003.tif]
